# Cohort profile: Greifswald approach to individualized medicine (GANI_MED)

**DOI:** 10.1186/1479-5876-12-144

**Published:** 2014-05-23

**Authors:** Hans J Grabe, Heinrich Assel, Thomas Bahls, Marcus Dörr, Karlhans Endlich, Nicole Endlich, Pia Erdmann, Ralf Ewert, Stephan B Felix, Beate Fiene, Tobias Fischer, Steffen Flessa, Nele Friedrich, Mariacarla Gadebusch-Bondio, Manuela Gesell Salazar, Elke Hammer, Robin Haring, Christoph Havemann, Michael Hecker, Wolfgang Hoffmann, Birte Holtfreter, Tim Kacprowski, Kathleen Klein, Thomas Kocher, Holger Kock, Janina Krafczyk, Jana Kuhn, Martin Langanke, Uwe Lendeckel, Markus M Lerch, Wolfgang Lieb, Roberto Lorbeer, Julia Mayerle, Konrad Meissner, Henriette Meyer zu Schwabedissen, Matthias Nauck, Konrad Ott, Wolfgang Rathmann, Rainer Rettig, Claudia Richardt, Karen Saljé, Ulf Schminke, Andrea Schulz, Matthias Schwab, Werner Siegmund, Sylvia Stracke, Karsten Suhre, Marius Ueffing, Saskia Ungerer, Uwe Völker, Henry Völzke, Henri Wallaschofski, Vivian Werner, Marek T Zygmunt, Heyo K Kroemer

**Affiliations:** 1 Department of Psychiatry and Psychotherapy, University Medicine Greifswald, Ellernholzstraße 1-2, Greifswald 17475, Germany; 2 Faculty of Theology, Ernst-Moritz-Arndt University Greifswald, Greifswald, Germany; 3 Institute for Community Medicine, University Medicine Greifswald, Greifswald, Germany; 4 DZHK (German Center for Cardiovascular Research), University Medicine Greifswald, Greifswald, Germany; 5 Department of Internal Medicine B, University Medicine Greifswald, partner site Greifswald, Greifswald, Germany; 6 Institute of Anatomy and Cell Biology, University Medicine Greifswald, Greifswald, Germany; 7 DZNE (German Center for Neurodegenerative Diseases), partner site Rostock/Greifswald, Greifswald, Germany; 8 Department of Internal Medicine, Pulmonary Diseases, University Medicine Greifswald, Greifswald, Germany; 9 Department of Internal Medicine A, University Medicine Greifswald, Greifswald, Germany; 10 Institute of the History of Medicine, University Medicine Greifswald, Greifswald, Germany; 11 Department of Health Care Management, Faculty of Law and Economics, Ernst-Moritz-Arndt University Greifswald, Greifswald, Germany; 12 Institute of Clinical Chemistry and Laboratory Medicine, University Medicine Greifswald, Greifswald, Germany; 13 Interfaculty Institute of Genetics and Functional Genomics, University Medicine Greifswald, Greifswald, Germany; 14 Institute for Microbiology, Ernst-Moritz-Arndt University Greifswald, Greifswald, Germany; 15 Department of Restorative Dentistry, Periodontology and Endodontology, University Medicine Greifswald, Greifswald, Germany; 16 Department of Pharmacology, University Medicine Greifswald, Greifswald, Germany; 17 Strategic Research Management, University Medicine Greifswald, Greifswald, Germany; 18 Institute of Medical Biochemistry and Molecular Biology, University Medicine Greifswald, Greifswald, Germany; 19 Department of Anaesthesiology and Intensive Care, University Medicine Greifswald, Greifswald, Germany; 20 Department of Philosophy, Ernst-Moritz-Arndt University Greifswald, Greifswald, Germany; 21 Institute of Biometrics and Epidemiology, German Diabetes Center, Leibniz Center for Diabetes Research at Heinrich Heine University Düsseldorf, Düsseldorf, Germany; 22 Institute of Physiology, University Medicine Greifswald, Greifswald, Germany; 23 Department of Neurology, University Medicine Greifswald, Greifswald, Germany; 24 Margarete Fischer-Bosch-Institute of Clinical Pharmacology, Stuttgart, Germany; 25 Department of Clinical Pharmacology, University Hospital, Tuebingen, Germany; 26 Institute for Bioinformatics and Systems Biology, Helmholtz Zentrum, München, Germany; 27 Resarch Unit of Protein Science, Helmholtz Zentrum, München, Germany; 28 Department of Obstetrics and Gynaecology, University Medicine, Greifswald, Germany; 29 Institute of History and Ethics of Medicine, Technical University Munich, Munich, Germany; 30 Institute of Epidemiology, Christian-Albrechts University Kiel, Kiel, Germany; 31 Department of Pharmaceutical Research, University Basel, Basel, Switzerland; 32 Department of Philosophy, Christian-Albrechts University Kiel, Kiel, Germany; 33 Bioinformatics Core, Weill Cornell Medical College, Doha, Qatar; 34 Institute for Ophthalmic Research, University of Tübingen, Tübingen, Germany; 35 Dean’s office, University Medicine Göttingen, Göttingen, Germany

**Keywords:** Personalized Medicine, Individualized Medicine, Epidemiology

## Abstract

**Background:**

Individualized Medicine aims at providing optimal treatment for an individual patient at a given time based on his specific genetic and molecular characteristics. This requires excellent clinical stratification of patients as well as the availability of genomic data and biomarkers as prerequisites for the development of novel diagnostic tools and therapeutic strategies. The University Medicine Greifswald, Germany, has launched the “Greifswald Approach to Individualized Medicine” (GANI_MED) project to address major challenges of Individualized Medicine. Herein, we describe the implementation of the scientific and clinical infrastructure that allows future translation of findings relevant to Individualized Medicine into clinical practice.

**Methods/design:**

Clinical patient cohorts (N > 5,000) with an emphasis on metabolic and cardiovascular diseases are being established following a standardized protocol for the assessment of medical history, laboratory biomarkers, and the collection of various biosamples for bio-banking purposes. A multi-omics based biomarker assessment including genome-wide genotyping, transcriptome, metabolome, and proteome analyses complements the multi-level approach of GANI_MED. Comparisons with the general background population as characterized by our Study of Health in Pomerania (SHIP) are performed. A central data management structure has been implemented to capture and integrate all relevant clinical data for research purposes. Ethical research projects on informed consent procedures, reporting of incidental findings, and economic evaluations were launched in parallel.

## Background

An increase in individualized diagnostic and therapeutic strategies is considered as means to improve patients’ treatment efficacy and safety. It might also allow for better individual outcome prediction and risk assessment. Moreover, individualized prevention and early intervention strategies are conceivable. On the economic side, a more efficient allocation of resources is pursued. Recently developed high throughput OMICs technologies are thought to enable more targeted diagnostic and treatment approaches. However, the issue of Individualized Medicine remains controversial. A lack of solid scientific evidence for the clinical utility of nearly all novel biomarkers is raised amongst the most common criticisms [1]. Furthermore, potential societal and ethical consequences of Individualized Medicine are sometimes not adequately addressed or even considered.

The research project “Greifswald Approach to Individualized Medicine” (GANI_MED) aims at implementing an increasing number of individualized diagnostic and therapeutic strategies in a university hospital and has its focus on cardiovascular, cerebrovascular and metabolic diseases and on ethical as well as economic aspects of Individualized Medicine. The main objectives are as follows:

•To recruit patient cohorts and to thoroughly examine these cohorts with standardized clinical measures

•To create a centralized bank of biologic specimens to support research needs and clinical care

•To link genetic and molecular analyses (‘omics’) to clinical data

•To assist investigators and encourage collaboration across different medical specialties.

To address these objectives a novel hospital information system extension that allows the efficient retrieval of routine clinical data for research purposes was necessary. At the same time, a large scale research database storing all data obtained in the hospital while fully conforming to high data safety standards needed to be established. Further, the potential ethical and economic implications of individualized medicine are addressed.

## Methods/design

### Study design

GANI_MED is conceptualized as cohort study with individual follow-ups. Starting with six main cohorts including patients with common diseases, three additional cohorts joined during the recruitment period. All subjects were regular patients of the university hospital except some of the patients with chronic renal failure who were also recruited from special treatment centers for dialysis. Study nurses or physicians from the participating departments enrolled the patients. All cohorts are thoroughly phenotyped using clinical methods, imaging technologies (ultrasonography, magnetic resonance imaging (MRI)), and OMICS approaches (Table 1). GANI_MED was approved by the ethics committee of the Medical Faculty of the Ernst-Moritz-Arndt University Greifswald. After complete description of the study to the patients, written informed consent is obtained. New infrastructures have been implemented (e.g. automated biobank, medical informatics) and additional research groups (e.g. research on ethical challenges) were founded. In the future, we plan to integrate all patients of the university hospital into the research data base, at a lower level of data assessment as compared to the present assessments in GANI_MED.

**Table 1:** **Examinations of the GANI**_**MED cohorts**

**Examination**	**Heart failure cohort**	**Cerebrovascular diseases cohort**	**Periodontal disease cohort**	**Renal disease cohort**	**Metabolic syndrome risk cohort**			**Fatty liver disease cohort**
					**Cardiovascular patients**	**Patients with depression**	PCOS* patients	
Clinical examination	×	×	×	×	×	×	×	×
Somatometric measures	×	×	×	×	×	×	×	×
Blood pressure	×	×	×	×	×	×	×	×
Number of teeth	×	×	×	×	×	×	×	×
ECG	×	×	-	-	×	-	-	-
Echocardiography	×	(×)	-	-	×	-	-	-
Echocardiography, diastolic function	×	-	-	-	×	-	-	-
Ankle-Brachial-Index	(×)	-	-	-	(×)	-	-	-
Pulse-wave-analysis	(×)	-	-	-	(×)	-	-	-
Endothelial function	(×)	-	-	-	(×)	-	-	-
Cardiopulmonary exercise	(×)	-	-	-	(×)	-	-	-
Carotid ultrasonography	-	×	×	-	-	-	-	-
Oral examinations	-	×	×	-	-	-	-	-
MRI of the head	-	×	×	-	-	×	-	-
Renal ultrasound	-	-	-	×	-	-	-	-
Bioelectrical impedance analysis	-	-	-	×	-	-	-	×
OGTT	-	-	-	-	×	×	×	-
Gynecological examinations	-	-	-	-	-	-	×	-
Uterus ultrasound	-	-	-	-	-	-	×	-
Liver ultrasound	-	-	-	-	-	-	-	×
Liver elastography	-	-	-	-	-	-	-	×
Pancreas ultrasound	-	-	-	-	-	-	-	×
Liver biopsy	-	-	-	-	-	-	-	×
MRI of the liver	-	-	-	-	-	-	-	×

*Polycystic Ovary Syndrome.

x = available, (x) = available only in subgroups, − = not available.

### Study population

We are recruiting six main cohorts with common cardiovascular, cerebrovascular or metabolic conditions: heart failure (expected n = 1200), stroke (expected n = 600), periodontal disease (expected n = 800), renal insufficiency (expected n = 400), metabolic syndrome (expected n = 1600), and fatty liver disease (expected n = 400). The official start of the patient recruitment was July 7, 2011. Three further cohorts (patients with sepsis, pulmonary diseases, and adverse medication effects) have been launched in the meantime (for detailed description of the cohorts see below).

### Study procedures

#### Standardized assessments and examinations

One of the key elements of GANI_MED is extensive quality control and standardization in the data acquisition. This is especially challenging as the data acquisition is part of routine care for patients, who are admitted to the university hospital Greifswald. Therefore, we have implemented an extended array of quality control measures regarding clinical phenotyping of our GANI_MED patients. These include computer-assisted standardized interviews to obtain the medical history (Table 2) and the medication taken by the patient. Standard operating procedures have been defined for each clinical examination, including the measurements of blood pressure, height, weight, hip circumference, waist circumference, and different ultrasonographic measures of the carotid arteries, kidney, liver, pancreas, and uterus. In contrast to established procedures in clinical practice, the medical staff has not only been trained for performing examinations, but also certified according to SHIP standards [2]. Numerous questionnaires are used to further assess the patients´ individual characteristics (Table 3). For example, in all GANI_MED cohorts childhood traumata (CTS, [3]) and current depressive symptoms (PHQ-9, [4]) are measured to enable an integrative approach to the impact of early traumatization and depression on the course and treatment response in somatically ill patients.

**Table 2:** **Content of the GANI**_**MED standardized medical history**

**Content**	Obligatory items*	**Special items†**
General Practitioner	1	8
Pulmonary and thoracic diseases	19	237
Cardiac conditions	7	-
Arterial hypertension	10	6
Coronary artery diseases	36	203
Inflammatory heart diseases	-	135
Heart failure	-	14
Cardiomyopathy	-	28
Valve defects	-	113
Cardiac arrhythmia	-	208
Catheter interventions	-	18
Peripheral artery occlusive disease	1	123
Aortic aneurysm	-	5
Thrombosis/pulmonary embolism	78	-
Gastrointestinal diseases	9	100
Liver/biliary tract diseases	29	80
Pancreatic diseases	14	131
Renal diseases	35	181
Metabolic diseases	42	8
Cancer	226	-
Stroke	36	632
Neurological conditions	-	204
Dermatological diseases	-	8
Inflammatory joint diseases	-	13
Gynecological conditions	-	270
Dental conditions	-	56
Mental disorders	44	40
Operations and therapies	8	-
Current conditions	124	45
Family history	-	16
Health behavior	142	3
Social history	8	12

*Standard in all cohorts †used only in some cohorts.

**Table 3:** **Self**-**report questionnaires in GANI**_**MED**

**Questionnaire**	**Heart failure cohort**	**Cerebrovascular diseases cohort**	**Periodontal disease cohort**	**Renal disease cohort**	**Metabolic syndrome risk cohort**			**Fatty liver disease cohort**
					**Cardiovascular patients**	**Patients with depression**	**PCOS patients**	
Patient Health Questionnaire – PHQ-9 [4]	×	×	×	×	×	×	×	×
Screening Questionnaire – SSQ/CID-S [5]	×	×	×	×	×	×	×	×
Screening of the “Stralsunder Lebensereignisliste” – SLE-S*	×	×	×	×	×	×	×	×
Childhood Trauma Screener – CTS [3]	×	×	×	×	×	×	×	×
The Aging Males´ Symptom rating scale – AMS [6]	-	-	-	-	×	×	×	-
International Index of Erectile Function (IEFF) [7]	-	-	-	-	×	×	-	-
KDQOL (Kidney Disease Quality of Life [8])/SF-36 [9]	-	-	-	×	-	-	-	-
Health Questionnaire EQ-5D [10]	-	-	-	-	-	-	-	×
EPIC-Nutrition-Questionnaire [11]	-	-	-	-	-	-	-	×
Minnesota Living With Heart Failure Questionnaire (MLHFQ) [12]	×	-	-	-	-	-	-	-
Health Cost of Heart Failure*	×	-	-	-	×	-	-	-
Health Cost of Renal Failure*	-	-	-	×	-	-	-	-

*Available upon request.

Moreover, data collection is constantly monitored for comprehensiveness, as well as for examiner variation and time trends due to unrequested methodical modification. Automated monitoring systems allow online recruitment monitoring and weekly feedbacks to the examiners.

By implementing these quality control measures we expect to obtain data that are of higher quality than is usually achieved in clinical contexts. The high data quality will not only allow us to use these data for scientific purposes in GANI_MED, but may also be beneficial for clinical practice.

#### Standardized medical history

The medical history, as it is obtained in clinical routine, is highly variable in terms of the information collected. In routine care, information collection varies with the qualification and time allocation of the examiner, the setting, the symptoms presented by the patients, the wording of questions, the sequence of questions, and the recording of answers. Each of these factors may affect the validity of the data, thereby limiting their exploitability for scientific purposes. Furthermore, the anamnesis is still often documented on paper rather than electronically, which further complicates its systematic re-use.

In order to standardize the medical history across the GANI_MED cohorts, we have developed a basic set of obligatory, standardized questions (Table 2), extended by optional cohort-specific special questions as part of a hierarchical structure of the interview. Answers to structured questions are directly coded in a portable computer. Each patient recruited for GANI_MED is interviewed as part of the medical history taking. The main areas covered by the interview are hypertension, cerebrovascular diseases, cardiac disorders, thrombosis, pulmonary embolism, peripheral vascular diseases, and gastrointestinal, hepatic, biliary, pancreatic as well as kidney diseases. Furthermore the history of metabolic diseases, stroke, cancer, mental disorders, surgery, current complaints, and current therapies as well as the social history and the history of health behaviors and lifestyle factors are recorded. In all, the cohort management IT solution for medical history covers a total of approximately 8000 electronic Case Report Form (eCRF) variables. The number of variables applying to a specific patient’s interview depends strongly on cohort membership and the course of the interview as questions are dynamically linked with answers given to previous questions.

#### Laboratory measures and biosampling

We have defined a specific set of laboratory parameters that is routinely measured for every patient on the day he/she is recruited for GANI_MED. These parameters include blood count, electrolytes, renal function parameters, liver function tests, blood lipids, inflammatory markers, and urinary markers (Additional file 1: Table S1). Furthermore, a comprehensive set of biomaterials including EDTA-plasma (6 ml), serum (5 ml), urine (6 ml), saliva (Salivette®, FA Sarstedt), as well as buccal and tongue smears are collected, assayed (in 850 μl cryo tubes) and stored in an automated biobank (STC12k-ULT KiWi Store, FA Liconic, Liechtenstein) for future laboratory analyses.

### Cohort-specific recruitments and examinations

Besides the core examination program, which includes the computer-assisted basic medical history and a comprehensive medication assessment, blood pressure and anthropometric measurements, a basic dental examination, and the common laboratory measurements, each cohort is characterized by additional examination procedures that are detailed below. The clinical characterization of patients in the different cohorts is summarized in Table 1.

#### Heart failure cohort

The research focus of this cohort is on chronic heart failure (HF). Patients with suspected or known HF due to systolic (HF with reduced ejection fraction = HFREF) or diastolic left ventricular dysfunction (HF with preserved ejection fraction = HFPEF) are recruited for this cohort. Within the HFREF cohort a specific focus is on patients with dilated cardiomyopathy [13]. HFPEF is considered in concordance with current recommendations [14]. The cohort specific phenotyping includes electrocardiography (ECG), transthoracic echocardiography and cardiopulmonary exercise testing in all patients. For selected subgroups additional examinations are performed such as cardiac MRI, myocardial biopsies (in patients with dilated cardiomyopathy), assessment of endothelial function by flow-mediated dilatation as well as pulse wave analysis and 24-h blood pressure measurement.

A follow-up for this cohort is intended based on further projects within the German Center for Cardiovascular Research (DZHK).

#### Cerebrovascular disease cohort

This cohort enrolls patients with acute ischemic stroke or transitory ischemic attacks (TIA) [15] who are admitted to the Stroke Unit of the Department of Neurology. All patients receive standard acute stroke treatment according to international guidelines [16,17] and a comprehensive diagnostic work-up including computer tomography (CT) or MRI, CT-angiography and/or ultrasonography of extracranial and intracranial arteries, echocardiography, Holter-ECG, and if necessary, laboratory tests for vasculitis and hypercoagulopathies to identify the underlying cause of stroke. Stroke subtypes are classified according to TOAST and ASCO criteria [18,19]. Affected vascular territories, sites of the infarction, and presence and extent of white matter lesions are recorded from brain imaging studies. Stroke severity is assessed with the National Institute of Health (NIH) stroke scale and the degree of disability is scored with the modified Rankin scale and the Barthel index on admission and at discharge. Cognitive function is assessed with the mini-mental state examination (MMSE) [20]. Furthermore, we record a comprehensive set of data on pre-hospital stroke management, symptoms at stroke onset, thrombolysis or clot retrieving procedures, secondary prevention therapy, in-hospital complications and concomitant disorders, as well as the presence of migraine according to International Headache Society criteria.

#### Periodontal disease cohort

This cohort includes patients with periodontal diseases. Periodontal disease is an inflammatory disease caused by an infection of the supporting tissue around the teeth which may lead to tooth loss if left untreated. Besides being a major cause of tooth loss, periodontal disease may be a risk factor for various systemic conditions and diseases [21,22]. We recruit patients, who have been on maintenance therapy for a long time, patients who were incompliant with maintenance and dropped out, and untreated newly admitted patients. Cohort specific examinations include MRI of the brain, ultrasonography of the carotid arteries including common carotid artery intima-media thickness measurement and assessment for atherosclerotic plaques [23]. The comprehensive dental examination comprises an interview with specific questions about oral hygiene, and standard dental check-ups, a gingival examination including measurements of pocket depth and attachment level, bleeding on probing, dental plaque (on six different sites per tooth). Biosamples include subgingival plaque, tongue smear, and stimulated saliva. Information on dental history and the periodontal treatment is retrieved from the patients’ charts and transferred into the GANI_MED data bank.

#### Renal and renovascular disease cohort

This cohort includes patients suffering from various forms of renal and renovascular disease. About two thirds of the cohort is represented by patients with end-stage kidney disease (ESKD) on dialysis. Patients are recruited in the dialysis centers of the KfH (Kuratorium für Dialyse und Nierentransplantation e.V.), a German nonprofit association, in Greifswald, Stralsund, and Demmin. About one third of the cohort with chronic kidney disease (CKD) is recruited in the division of nephrology (Department of Internal Medicine A). CKD is assessed by estimating glomerular filtration rate from serum creatinine levels using the Modification of Diet in Renal Disease (MDRD) formula [24], and by measuring the albumin-to-creatinine ratio in spot urine [25]. Cohort specific examinations include an ultrasonographic examination of the kidneys, a microscopic urinary sediment analysis, a standard questionnaire (Kidney Disease Quality of Life – KDQOL [8]/SF-36 [9]), quantitative determination of the activity of a defined panel of proteases in the blood plasma, and the isolation of RNA and exosomes from urine. It should be noted that about 20% of the patients recruited by other GANI_MED cohorts exhibit CKD as a comorbidity (defined by an estimated glomerular filtration below 60 ml/min/1.73 m^2^).

#### Metabolic syndrome risk cohort

Subjects that are on an increased risk for or actually suffer from metabolic syndrome are eligible for this cohort. Metabolic syndrome is defined as fulfilling three or more of the following five criteria: (1) Abdominal obesity: waist circumference ≥94 cm or ≥80 cm for men and women, respectively (2) elevated blood pressure: ≥130/85 mmHg or self-reported antihypertensive medication; (3) elevated non-fasting glucose: ≥8.0 mmol/l or diabetic medication (ATC code A10); (4) elevated triglycerides: ≥2.3 mmol/l or lipid-modifying medication (ATC code C10AB or C10AD); (5) and reduced high-density lipoprotein (HDL) cholesterol: <1.03 mmol/l or <1.29 mmol/l for men and women, respectively. This definition has been proposed by the National Cholesterol Education Program/Adult Treatment Panel III (NCEP/ATP III) [26], and the International Diabetes Federation [27] updated with minor modifications by the American Heart Association and the National Heart, Lung, and Blood Institute [28] using non-fasting blood samples [29,30].

All patients fulfilling the criteria for metabolic syndrome without meeting the diagnosis of diabetes mellitus receive an oral glucose tolerance test.

In order to assess possible hormonal disturbances related to distinct comorbid conditions associated with the metabolic syndrome, this cohort consists of three sub-cohorts:

•Patients with the primary diagnosis of mental disorders are recruited from the day clinic of the Department of Psychiatry and Psychotherapy. All patients of the day clinic are invited to take part in the GANI_MED project. The main diagnoses of these patients are depressive disorders (90%), anxiety and somatoform disorders, trauma-related disorders and personality disorders.

•Women with polycystic ovary syndrome (PCOS) are recruited at the Department of Obstetrics and Gynaecology. PCOS is a highly prevalent heterogeneous disease affecting 1 in 5 women in reproductive age. PCOS is characterized by clinical or biochemical androgen excess and/or anovulation, and polycystic ovaries on ultrasound. PCOS increases the risk of insulin resistance, type 2 diabetes mellitus, visceral obesity, cardiovascular disease, infertility, and depression. The etiology of PCOS remains unclear and the highly individual phenotype makes diagnosis difficult. It seems that PCOS is caused by a combination of environmental and genetic factors. New and individualized strategies are needed for an early diagnosis and age adjusted treatment. A comprehensive characterization of clinical phenotypes, clinical risk profiles as well as the identification of biological correlates from genome, proteome, transcriptome, and metabolome data is performed.

•Patients with the metabolic syndrome and prevalent cardiovascular diseases (e.g., coronary artery disease, myocardial infarction, hypertensive heart disease) and at least three metabolic syndrome criteria are recruited from the Department of Internal Medicine B/Cardiology. For specific phenotyping of selected subjects of this cohort, methods that are also applied in the cardiovascular cohort are being used (ECG, transthoracic echocardiography, cardiopulmonary exercise testing, assessment of endothelial function, pulse wave analysis, 24-h blood pressure measurement).

#### Fatty liver disease cohort

This cohort includes patients with non-alcoholic fatty liver disease (NAFLD). Screening of patients is performed by transabdominal ultrasound. In case of a hyperechogenic pattern of the liver parenchyma in comparison to the kidney patients are recruited for the study. The diagnosis of NAFLD is based on a positive ultrasound examination in conjunction with a NAFLD score of < −1.455 to <0.675 (low-cut-off: estimated sensitivity 90%, specificity 60%) [31,32] or an APRI score of >0.5 [33]. Patients with at least one of the following conditions are excluded: Liver cirrhosis, alcohol abuse or consumption >30 g alcohol per day, pulmonary hypertension, dilated cardiomyopathy with ejection fraction < 30%, autoimmune hepatitis, viral hepatitis, Wilson disease (hepatolenticular degeneration), hemochromatosis, α-1-antitrypsin deficiency, amyloidosis, malignoma of the liver, primary biliary cirrhosis or primary sclerosing cholangitis. A cohort specific interview has been developed addressing relevant information for NAFLD disease. Cohort specific examinations include ultrasonography and real time tissue elastography of the liver using a Preirus device (Hitachi, Tokyo, Japan). In a fraction of patients liver biopsy is obtained, provided that it is clinically indicated. The liver elasticity score is done on 10 images with compression values of 3–4 on a scale elastography of the liver. Bioelectrical body impedance analysis is performed using Nutriguard M (Data Input GmbH, Darmstadt, Germany). R (resistance) and Xc (reactance) are measured applying electric currents of 800 mA at 50 kHz. Source and sensor electrodes are placed on the dorsum of hand and foot of the dominant body side. A quantitative chemical shift-encoded MRI to quantify hepatic steatosis is performed [34]. Further, a dietary questionnaire and a validated score to assess physical activity [11] are administered as well as the EQ5D [10] for the assessment of Quality of Life.

A cohort of patients with acute and chronic pancreatitis is recruited in parallel for comparison of phenotypic, genetic and metabolic characteristics as well as biomarkers.

#### Cohort of adverse medication effects

This cohort includes all patients who are admitted to the Departments of Internal Medicine due to severe adverse drug reactions (ADR). All non-elective hospital admissions are screened for ADR by a clinical pharmacologist. In patients with suspected ADR, a detailed history (demographical patient data, admission diagnosis, comorbidities, risk factors, drugs taken prior to onset of reaction) is obtained and results of clinical and laboratory tests as well as ADR description (e.g., severity, course, outcome) are gathered. Patients receiving cancer chemotherapy are excluded.

Outcomes of ADRs are classified according to International Conference of Harmonization (ICH) guidelines. The assessment of ADR severity is based on the adverse drug reaction reporting system described by Hartwig et al. [35]. Classification of ADR types is performed according to Edwards & Aronson [36]. A standardized causality assessment of each drug taken before the hospital admission is made using the Begaud algorithm [37]. Preventability of ADRs is assessed by standardized criteria (e.g., non-adherence, dose-related problems, relevant drug-drug-interactions). All ADRs are reviewed in a quality assurance and clinical plausibility check by a second clinical pharmacologist.

Incidences of ADRs will be estimated by the number of events per 1000 treated patients per year using prescription data provided by health insurances. Blood samples for genotyping are collected from all patients with ADR in order to identify genetic risk factors using modern technologies of pharmacogenomic approaches.

Recently launched cohorts:

#### Cohort of pulmonary diseases

The aim of this cohort is a thorough characterization of the individual cardiovascular risk factor profile and the co-morbidities in patients with chronic obstructive pulmonary disease (COPD). This cohort is set up to clarify the association of various co-morbidities with cardiopulmonary capacity, quality of life, and ultimately the mortality risk of patients. In-patients with COPD (defined by obstructive ventilation problems and clinical symptoms) are included in this cohort in accordance with current guidelines [38]. We aim at recruiting 300 patients. An extensive medical history taking, clinical examination, somatometric measures, blood pressure, assessment of oral status (number of teeth), ECG, and transthoracic echocardiography are performed in each patient. The cohort specific phenotyping includes a six-minute walk test (6-MWT) [39], lung function analysis, blood gas analysis, and polysomnography. In a selected subgroup, additional measurements will be performed including MRI scanning of thorax, heart, and hamstring muscle.

Examinations are complemented by specific questionnaires regarding sleep-associated symptoms (Berlin Questionnaire [40], Epworth Sleepiness Scale [41], Index of the Severity of Insomnia (ISI) [42], Pittsburgh Sleep Quality Index (PSQI) [43], and a COPD-specific Assessment Test (CAT) [44]). Hence the generation of established prognostic indices (Bode-Index [45], ADO-Index [46]) and recent COPD graduations [47] is possible. All COPD patients are offered participation in the long-term monitoring study COSYCONET that will eventually include about 3,000 COPD.

#### Cohort of sepsis

Sepsis and septic shock still contribute significantly to in-hospital mortality, in particular in intensive care units, despite major progress in hygiene and antibiotic treatment [48]. To date, all major trials trying to repeat promising experimental approaches from the bench and animal studies in septic patients have failed. It remains unclear which combination of patient characteristics may determine the clinical course of the condition, and therefore the survival of the patients [49]. Patients are enrolled in the cohort, based on established sepsis criteria. All patients are followed up for three consecutive time points. The major aim of this cohort is to identify clinical and OMICS-markers that allow for prediction of the sepsis course and may guide individual treatment decisions.

### Medical informatics and data management

**Figure 1:**
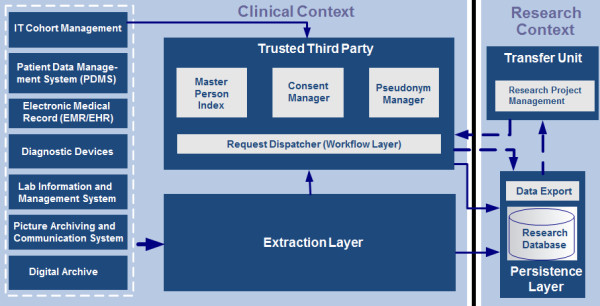
Architecture and basic elements of the GANI_MED research IT-platform.

Individualized Medicine requires complex platforms to acquire and manage data sets for evidence-based research, including data transformation, normalization, and long term storage. A high degree of automation is essential to efficiently acquire, integrate and apply Use & Access rules to data from a variety of sources, including electronic Case Report Forms (eCRF), a patient’s health record, diagnostic devices such as MRI, sonography, and ECG, or sources for secondary data like a population register, health insurance or a patient’s family doctor [50]. Compliance with public and local regulations of data privacy as well as information security has to be ensured. Scalability and high availability of at least those tools that are used to record data is another key aspect to consider. Applicability of the Medical Device Directive (MDD) of the EU, together with country-specific regulations, must be carefully considered when designing and operating such platforms and tools. The informed consent is a basic requirement for the scientific use of patient data. GANI_MED adopts a modular consent design that allows patients to agree or disagree to particular modules in an opt-in model (e.g., use of data, biomaterials, information about incidental findings, contact in the future). An independent Trusted Third Party as institutional as well as technical instrument is implemented to centrally manage the informed consents of all GANI_MED participants. Furthermore, the Trusted Third Party ensures unique and platform-wide identification of persons, independent from data source and local identifiers. Another unique identifier (Master Person Index) is used as a link between all source-specific identifiers and identities. A probabilistic approach allows tolerating minor spelling mistakes or missing information; ambiguous matches are resolved manually at a later point of time while data can be recorded continuously. A conservative approach is taken in case of an ambiguous match: two identifiers are used, which can be combined once both identities have been approved as belonging to a single person. Furthermore the Trusted Third Party generates and manages all pseudonyms used throughout the platform. The tools used in GANI_MED can operate on almost arbitrary alphabets (numeric, characters, special characters, mixes). Parameters can be adjusted to optimize for error correction or error recognition. Use & Access of data is managed through a transfer unit. When transferring data to a research project, all pseudonyms are made project-specific pseudonyms to prevent aggregation of larger data sets across a number of projects. Figure 1 shows the high-level architecture of the GANI_MED research IT-platform.

Cohort management is done using Java-based offline-capable eCRF software on mobile clients, so-called Mobile Clinical Assistants [51]. These are used to record patients’ personal data, document the informed consent, and generate a cohort-specific number of dynamic eCRF-based data, including lab order IDs and similar links to external sources. All data are synchronized with a server application which functions as one of the data sources (left hand side of Figure 1). Currently, the cohort management is used for nine GANI_MED cohorts, implementing 225 electronic forms, more than 8,000 variables and 200,000 data sets. The Extraction Layer of the platform accounts for separating personal (i.e., identifying) data from medical data and performs source-specific transformations into a platform-internal unified data format for further processing. Presently (March 3, 2014), the platform operates on 18 million data points for 3,535 persons participating in GANI_MED. The three functional blocks of the Trusted Third Party have successfully been re-used and adapted for other projects such as the German Center for Cardiovascular Research (DZHK) or The National Cohort in Germany, a large representative population cohort that will start recruitment of 200,000 subjects in 2014.

### Storage facility for biosamples

An automated biobank (STC12k-ULT KiWi Store, FA Liconic, Liechtenstein) was installed to store and handle up to 500,000 cryo tubes of 1 ml volume at a constant temperature of -80°C. Nineteen cryo tubes with biomaterials including serum (N = 5), EDTA-plasma (N = 5), urine (N = 5), saliva (N = 2), and DNA (N = 2) are processed and stored for each patient.

### Deep phenotyping of patients with genomics and functional genomics approaches

Deep phenotyping of patients with genomics and functional genomics approaches provides molecular information at unprecedented detail that will allow much more in-depth description of the (patho)physiological status of a patient. The OMICs data generated at different levels (genomics, transcriptomics, proteomics, and metabolomics) will likely allow for better descriptions of health and disease states [52]. Within GANI_MED, standardized workflows for the OMICs characterization of biosamples will be developed. Currently, genomic variants are analyzed for 1,800 patients with the aid of the HumanCoreExome + v1.1-Psych Array. For a subset of 1,200 patients, whole blood expression data are generated using Illumina’s HT-12 bead chips. These data will be complemented with proteomics and metabolomics data using established workflows.

Metabolomics provide comprehensive snapshots of the metabolome of body fluids such as plasma, urine or saliva. Within GANI_MED, high-throughput metabolomics analyses mainly based on 1H nuclear magnetic resonance (NMR) spectroscopy, a non-destructive analysis with minimal preparation requirements, are performed. NMR spectroscopy provides robust and reproducible measurements. Mass spectrometry (MS) with high analytical sensitivity will be used for additional detailed studies.

Integrated analyses of these data for associations with clinical and subclinical phenotypes will be performed by the bioinformatics groups of the GANI_MED consortium.

### Economic aspects of Individualized Medicine

The health economic analysis contributes to the comprehensive assessment of advantages and disadvantages of Individualized Medicine by comparing costs of innovative diagnostics with their effectiveness. One focus lies on the performance of an economic evaluation of the predictive value of genetic and non-genetic biomarkers to forecast health care costs. The analysis which is based on SHIP data, correlates genetic and non-genetic biomarkers with the health seeking behavior and/or health care expenditure [53]. For instance, we demonstrated that rs738409 (GG) in PNPLA3 is a significant predictor of hospital admissions for gallstone complaints. Secondly, we perform cost analyses of severe drug-related side effects as they are a major cause of hospital admissions and cause a large fraction of the global health care expenditure. Thus, it is crucial to understand the costs and benefits of specific treatment options or diagnostic tests such as genetic testing. We could show that the immense costs caused by intestinal bleeding as a side effect of phenprocoumon therapy are so high that a specific genetic test for every patient taking this drug would be cost-effective [54]. Thirdly, economic analyses evaluate the cost-effectiveness of specific therapeutic interventions of Individualized Medicine [55].

### Ethical research

One general strategy in GANI_MED is the recruitment of patients during ongoing clinical routine care. This means that hospitalized patients are being asked to make their clinical data and biomaterial available for scientific research. Thus, one major challenge for the ethical working group was to develop an appropriate information concept to enable patients to give their informed consent. This informed consent procedure covers the multilevel assessment of personal, clinical and biological data as well as the storage and use of data and the biobanking. All informed consent procedures are carried out according to standards of law, data protection, and research ethics. This includes the preparation of the information and consent forms, an ongoing training for the GANI_MED staff as well as the design and implementation of workflows and standard operation procedures regarding all ethically sensitive processes in GANI_MED [56,57].

Some GANI_MED cohorts also include non-treatment related study examinations. Ethical challenges emerge, if incidental findings, that might be relevant for the health of the participants, occur in these cases. Their management does not follow clinical routine care. Especially when the participant does not suffer from any symptoms of illness researchers need to weight potential harm against potential benefit of disclosure of medical findings for the participant. The benefit might be the early detection and treatment of pathological states whereas invasive diagnostic procedures as well as psychological distress associated with the disclosure of incidental findings represent relevant negative consequences.

The non-clinical use of imaging methods like the whole body MRI or genome-wide data represents an ethical and psychological challenge in case of incidental findings.

To address this issue, one of our ethical research projects specifically focuses on the disclosure of incidental findings. The project applies methods from empirical social research to examine how the disclosure of incidental findings will affect the participants within medical research projects. This is done within the framework of the population-based SHIP study [2] at the University Medicine Greifswald with a special focus on the whole body MRI. The view of the participants is inferred from their answers to questionnaires and interviews with SHIP participants who were informed about their incidental MRI findings [58-60].

### Reference groups for case–control comparisons

The University Medicine Greifswald has a long tradition in community-based research. SHIP is a large prospective cohort study that was implemented in 1997 (SHIP-0) to investigate a broad spectrum of health-related parameters in West Pomerania, a rural area in the north-east of Germany with a relatively short life expectancy as compared to that in the average German population [2]. Over the past 15 years, the population-based study SHIP has evolved as cohort study with one of the most comprehensive phenotype assessments worldwide covering the follow-up waves SHIP-1, SHIP-LEGEND and SHIP-2. From 2008 to 2012 a new, independent sample called SHIP-TREND was recruited (n = 4420). Both representative samples from the general population serve as reference sample for case–control comparisons with the GANI_MED patient cohorts. The university hospital Greifswald is the only major hospital in the area. The SHIP sample was derived from the same geographic area, ensuring that patient cohorts and control groups originate from the same population.

### Access to the GANI_MED data

External collaborations are welcome. For the coordination of research projects based on the GANI_MED data, a request and transfer process has been established that largely follows the procedure established in SHIP. A web-based request form will be available soon. Meanwhile interested researchers may download a form at the data transfer unit (http://www.community-medicine.de) and send the completed document to grabeh@uni-greifswald.de. Together with collaborators at the University Medicine Greifswald we will select the necessary variables and complete the application. Once the application has been approved, a contract is concluded between the external applicant and the Research Network of Community Medicine.

## Discussion

We will apply hypothesis-generating and hypothesis-testing approaches with additional replication and validation steps. Those strategies include the testing of associations in SHIP-0 and the replication of the findings in SHIP-TREND. In a translational step, associations will be transferred to the clinical samples of GANI_MED to check for their validity in severely ill patients. On the other hand, associations primarily identified in clinical setting can be tested for their relevance in the general population. Identified or suspected biomarkers can be tested for their predictive value for incidental diseases in the longitudinal waves of SHIP. Clinical samples from GANI_MED can be stratified based on clinical and biological markers and investigated for differential outcomes, courses, and treatment responses. Biological mechanisms will be addressed by biological systems approaches and other experimental approaches such as cell culture and animal studies, which are performed in collaboration with our local, national or international collaborators.

### Highlights

As data collection and OMICS analyses in GANI_MED are still ongoing, research is focusing on the analyses of SHIP and laboratory data to generate new hypotheses for translational research. Research in the field of hormones like testosterone, prolactin and IGF-1 as individual biomarkers and putative predictors of clinical outcomes (e.g., hypertension, depression, inflammation) has been especially successful [61-66]. Novel biomarkers like angiopoietin-2, Tie-2 [67], homoarginine and asymmetric dimethylarginine (ADMA) [68] are under investigation. Metabolites have been analyzed by NMR and mass spectroscopy and are currently tested for their association and predictive properties in various diseases [69]. Strong associations between genetic variations and metabolites have been discovered [70]. Myocardial gene expression profiles have been used to successfully predict the treatment response to immunoadsorption therapy in patients with dilated cardiomyopathy [71]. The important interface between oral and systemic health is increasingly addressed in GANI_MED [72], especially the inflammatory effects of periodontitis [73]. Growth factor receptor-mediated signaling is meanwhile recognized as a complex signaling network. The interactome of the epidermal growth factor receptor (EGFR) was identified and quantified. The newly developed Cytoscape plugin ModuleGraph facilitated the extension and functional investigation of this network [74]. A short 5-item questionnaire for routine clinical assessment of childhood abuse and neglect has been developed and validated [3,75]. Thus, the effects of gene-environment interactions in somatically burdened patients can be investigated [76,77].

A standardized protocol for fat quantification in fatty liver disease on liver MRI has been developed and validated within this cohort [34,78,79].

## Conclusions

As new levels of biological markers (e.g. genomics, transcriptomics, metabolomics, proteomics) are becoming increasingly available at a decreasing level of costs, medical research is obliged to explore the new insights derived from new levels of biological information for their practical use in medicine. We need to explore the new biological markers to discover, validate and implement novel biomarkers that allow for better and individualized diagnostics and therapies. We have managed to implement an extensive infrastructure for the collection of standardized clinical data, laboratory analyses, technical examinations and biobanking for routine patients in our university hospital. A comprehensive informed consent procedure was established that enables re-contacting and follow-up of the patients. This effort should be pursued jointly with academic partners as well as health care and biotechnical companies. In the near future, we plan to build up an IT-based network with local general practitioners and specialists for the communication of relevant health information. For the use and interpretation of novel biomarkers local general practitioners and specialists will need some guidance and support which we will provide in a collaborative way.

## Competing interests

The authors declare that they have no competing interests.

## Authors´ contributions

HJG, HA, MD, KE, NE, SBF, MH, WH, MML, HM, MN, KO, WR, MS, WS, KS, MU, UV, HV and HKK were involved in the conception and design process of the GANI_MED study. HJG, TB, MD, KE, PE, RE, SF, NF, MGS, EH, RH, WH, TKo, ML, MML, WL, RL, JM, KM, MN, KS, US, UV, HV, VW and MTZ drafted the manuscript. HA, SBF, BF, TF, MGB, CH, MH, BH, TKa, KK, HK, JKr, JKu, UL, HM, KO, RR, CR, MS, WS, SS, KS, SU and HKK revised it critically. Additionally, HJG, MD, KE, NE, BF, CH, WH, BH, TKo, UL, MML, KM, MN, US, AS, WS, SS, SU, HW, and MTZ have made substantial contributions to the acquisition of data. HJG, MD, NE, RE, SBF, BF, TF, SF, TKa, KK, TKo, JKu, RL, HM, RR, US, AS, SS, KS, and UV were responsible for analysis and interpretation of data. NF, RH, MN, HW, and MTZ were in charge of the new automated Biobank. TB, CH, WH, and JKr were responsible for the set-up of the IT infrastructure. TKo, RL, AS, and HV have contributed to quality control of the data. HA, PE, TF, MGB, CH, WH, ML, and AS implemented the informed consent procedure. HJG, KE, NE, NF, MGS, EH, MH, TKa, TKo, JM, KM, MN, KS, UV, and HW analyzed OMICS data. HJG, HK, WL, CR, HW, VW, and HKK were in charge of the overall project coordination. All authors read and approved the final manuscript.

## Supplementary Material

Additional file 1: Table S1 Laboratory parameter in GANI_MED. Click here for file
