# Supplementary material for: Cohort profile: Greifswald approach to individualized medicine (GANI_MED)
Source: J Transl Med. 2014 May 23;12:144. doi: 10.1186/1479-5876-12-144 (PMC4040487; doi:10.1186/1479-5876-12-144)
Supplement: Additional file 1: Table S1 — Laboratory parameter in GANI_MED. [file 1479-5876-12-144-S1.pdf]

Supplementary table 1: Laboratory parameter in GANI_MED

Parameter	Unit	Lower Reference Limit	Upper Reference Limit
**EDTA plasma**			
Leucocytes	Gpt/l	4,3	10,0
Erythrocytes (Erys)	Tpt/l	4,2	6,2
Hemoglobin	mmol/l	7,4	11,2
Hematocrit		0,35	0,51
MCV	fl	80	95
MCH	fmol	1,68	2,00
MCHC	mmol/l	18,5	22,5
Ery distribution	%	11,0	16,0
Range of ery distribution	fl	37	54
Thrombocytes	Gpt/l	140	440
Mean platelet volume	fl	9,0	13,0
Range of platelet distribution	fl	9,0	17,0
Prothrombin time (Quick)	%	70	130
HbA1c	%	0	6,5
**Citrate plasma**			
INR		1,0	1,2
aPTT	s	25	33
**Heparin plasma**			
Sodium	mmol/l	135	145
Potassium	mmol/l	3,7	5,1
Calcium	mmol/l	2,20	2,65
Phosphate	mmol/l	0,60	1,60
Glucose	mmol/l	3,9	6,4
Creatinine	µmol/l	58	127
Urea	mmol/l	2,5	6,4
Uric acid	µmol/l	155	428
Total Cholesterol	mmol/l		<6,0
HDL-Cholesterol	mmol/l	>1,03	
LDL-Cholesterol	mmol/l	0	3,34
Triglycerides	mmol/l	0	1,9
ALAT (GPT)	µkatal/l	0	0,77
ASAT (GOT)	µkatal/l		<0,59

Gamma-GT	µkatal/l	0	0,96
Bilirubin total	µmol/l	0	17,0
Lipase	µkatal/l	1,59	6,36
CRP	mg/l		<5,0
TSH	mU/l	0,25	2,11
Albumin	g/l	34	50
Protein	g/l	65	85
**Urine**			
Specific weight	kg/l	1,005	1,030
pH		4,5	7,5
Leucocytes	number/µl	0	10
Nitrite	positive/negative	negative	negative
Protein	g/l	0	0,2
Glucose	mmol/l	0	2
Ketone	mmol/l	0	0,2
Urobilinogen	µmol/l	0	10
Bilirubin	µmol/l	0	5
Erys/Blood	number/µl	0	5
U-Creatinine	mmol/l	3,0	11,5
U-Albumin	mg/l	2	35
